# A support vector machine-based cure rate model for interval censored data

**DOI:** 10.1177/09622802231210917

**Published:** 2023-11-08

**Authors:** Suvra Pal, Yingwei Peng, Wisdom Aselisewine, Sandip Barui

**Affiliations:** 1 Department of Mathematics, 12329 University of Texas at Arlington, TX, USA; 2 Department of Public Health Sciences, Queen’s University, Kingston, ON, Canada; 3 Quantitative Methods and Operations Management Area, Indian Institute of Management Kozhikode, Kozhikode, KL, India

**Keywords:** Support vector machine, multiple imputation, sequential minimal optimization, mixture cure rate model, expectation–maximization algorithm

## Abstract

The mixture cure rate model is the most commonly used cure rate model in the literature. In the context of mixture cure rate model, the standard approach to model the effect of covariates on the cured or uncured probability is to use a logistic function. This readily implies that the boundary classifying the cured and uncured subjects is linear. In this article, we propose a new mixture cure rate model based on interval censored data that uses the support vector machine to model the effect of covariates on the uncured or the cured probability (i.e. on the incidence part of the model). Our proposed model inherits the features of the support vector machine and provides flexibility to capture classification boundaries that are nonlinear and more complex. The latency part is modeled by a proportional hazards structure with an unspecified baseline hazard function. We develop an estimation procedure based on the expectation maximization algorithm to estimate the cured/uncured probability and the latency model parameters. Our simulation study results show that the proposed model performs better in capturing complex classification boundaries when compared to both logistic regression-based and spline regression-based mixture cure rate models. We also show that our model’s ability to capture complex classification boundaries improve the estimation results corresponding to the latency part of the model. For illustrative purpose, we present our analysis by applying the proposed methodology to the NASA’s Hypobaric Decompression Sickness Database.

## 1. Introduction

Ordinary survival analysis techniques such as the proportional hazards (PH) model, the proportional odds (PO) model or the accelerated failure time (AFT) model are concerned with modeling censored time-to-event data by assuming that every subject in the study will encounter the primary event of interest (death, relapse, or recurrence of a disease, etc.). However, it is not appropriate to apply these techniques to situations where a portion of the study cohort does not experience the event, for example, clinical studies involving low fatality rate with death as the event. It can be argued that if these subjects are followed up sufficiently beyond the study period, they may face the event due to some other risk factors. Therefore, these subjects can be considered as cured with respect to the event of interest. The survival model that incorporates the effects of such cured subjects is called the cure rate model. Remarkable progress in medical sciences also necessitate further exploration in the cure rate model where estimating the cure fraction precisely can be of great importance.

Introduced by Boag^1^ and exclusively studied by Berkson and Gage,^2^ the mixture cure rate model is perhaps the most popular cure rate model.^3^ If $T^{*}$ denotes the lifetime of a susceptible (not cured) subject, then, the actual lifetime T for any subject can be modeled by

(1) $$T=JT^{*}+(1-J)\infty$$

where J is a cure indicator with J=0 if an individual is cured and J=1 otherwise. Furthermore, considering $S_{p}(t)=P(T>t)$ and $S_{u}(t)=P(T^{*}>t)$ as the respective survival functions corresponding to T and $T^{*}$ , we can express

(2) $$S_{p}(t)=(1-\pi )+\pi S_{u}(t)$$

where π=P(J=1) . The latency part $S_{u}(t)=S_{u}(t|x)$ and the incidence part π=π(z) are generally modeled to incorporate the effects of covriates $x=(x_{1},\ldots ,x_{p}{)}^{T}$ and $z=(z_{1},\ldots ,z_{q}{)}^{T}$ for any integers p and q . Note here that x and z may share the same covariates.

The properties of the mixture cure rate model with various assumptions and extensions are explored in details by several authors. Modeling lifetime of the susceptible individuals have been studied extensively. For example, a complete parametric mixture cure rate model is studied by assuming homogeneous Weibull lifetimes and logit-link to the cure rate.^4,5^ Semiparametric cure models with PH structure of the latency has also been studied extensively.^6–8^ Generalizations to semiparametric PO,^9,10^ AFT,^11–13^ transformation class,^14^ and additive hazards^15^ under mixture cure rate model were also investigated with various estimation techniques and model considerations.

On the other hand, the incidence part π(z) is traditionally and extensively modeled by sigmoid or logistic function

(3) $$\pi (z)=\frac{\exp\;(z^{*T}\beta )}{1+\exp\;(z^{*T}\beta )}$$

where $\beta =(\beta_{0},\beta_{1},\ldots ,\beta_{q}{)}^{T}$ and $z^{*}=(1,z^{T}{)}^{T}$ .^4,6,7,16–20^ As observed in the case of logistic regression, the logistic model works well when subjects are linearly separable into the cure or susceptible groups with respect to covariates. However, problem arises when subjects cannot be separated using a linear boundary. Other options to model the incidence include assuming a probit link function ( $\Phi^{-1}(\pi (z))=z^{*T}\beta$ ) or a complementary log-log link function ( $\log\;[-\log\;(1-\pi (z))]=z^{*T}\beta$ ), where Φ is the cumulative distribution function of the standard normal distribution.^21–23^ However, similar to the logit link (3), these link functions do not offer nonlinear separability and are not sufficient to capture more complex effects of z on the incidence. Non-parametric strategies, for example, the generalized Kaplan–Meier estimate at maximum uncensored failure time^24^ to estimate the incidence part π(z) and the modified Beran-type estimator^25^ to estimate the latency part in a mixture cure model, are also considered in the literature. Again, applying these strategies to multiple covariates can be challenging. Other non-parametric spline-based mixture cure models are capable of capturing complex patterns in the data, but they do not perform well when there are a large number of covariates with complicated interaction terms, which is a serious drawback.^26,27^ Therefore, there exists necessity to identify a group of classifiers which would be able to model the incidence part more effectively by allowing nonlinear separating boundaries between the cured and non-cured subjects.

To this end, the support vector machine (SVM) could be a reasonable choice.^28^ Introduced by Cortes and Vapnik,^29^ the SVM is a machine learning algorithm that finds a hyperplane in multidimensional feature space that maximizes the separating space (margin) between two classes. The main advantage of the SVM is that it can separate nonlinear inseparable data by transforming it to a higher dimensional space using kernel trick. Consequently, this classifier is more robust and flexible than logit or probit link functions. Given the availability of different machine learning algorithms,^30^ we propose to use SVM-based techniques in this article mainly because SVM is based on the kernel trick and hence it is possible to design or fuse kernels to improve performance. Furthermore, SVM uses a subset of training points in the decision function, which makes it memory efficient. Additionally, the execution time for SVM is expected to be less when compared to other classifiers such as artificial neural networks (NNs). Recently, Li et al.^31^ studied the effect of the covariates on the incidence π(z) by implementing the SVM. The new mixture cure rate model is seen to outperform existing logistic regression-based mixture cure rate model especially in the estimation of the incidence, and performs well for nonlinearly separable classes. However, the authors only considered data under non-informative right censoring mechanism.

Unlike right-censored data,^32,33^ interval-censored data occur for a study where subjects are inspected at regular intervals, and not continuously.^34–36^ If a subject experiences the event of interest, the exact survival time is not observed and is only known that the event has occurred between two consecutive inspections. Interval-censored data marked by cure prospect are often observed in follow-up clinical studies (cancer biochemical recurrence or AIDS drug resistance) dealing with events having low fatality and patients monitored at regular intervals.^37,38^ As in the case of right-censored data, some subjects may never encounter the event of interest, and are considered as cured. Mixture cure models for interval censored data have been studied and several estimation methods were proposed for both semiparametric and non-parametric set-ups.^39–43^ Motivated by the work of Li et al.,^31^ we propose to employ the SVM-based modeling to study the effects of covariates on the incidence part of the mixture cure rate model for survival data subject to interval-censoring. In addition, we compare our model not only with the logistic regression-based mixture cure model but also with the spline regression-based mixture cure model, which is also capable of capturing complex effects of z on π(z) . Note that we use the spline method only in the incidence part of the mixture cure model. To apply the spline model to a classification problem where the response variable is qualitative in nature (as in the case of this article), we approximate the log-odds with a smoothing function. In this article, we consider the thin plate spline as the smoothing function which can accommodate multiple predictor variables and also allows the degrees of freedom and the basis function to be selected automatically from the mathematical statement of the smoothing.^44,45^ In particular, to capture nonlinear effect of z on π(z) we have

(4) $$\pi (z)=\frac{\exp\;(g(z))}{1+\exp\;(g(z))}$$

where g(z) is a smooth function which is estimated using a thin plate spline by

(5) $$\hat{g}(z)=\sum_{i=1}^{n}\tau_{i}\eta_{mq}(||z-z_{i}||)+\sum_{j=1}^{M}\alpha_{j}\phi_{j}(z).$$

In (5), n is the total number of observations, m is such that 2m>q , τ and α are vectors of coefficients to be estimated, and τ is subject to the linear constraints $T^{T}\tau =0$ with $T_{ij}=\phi_{j}(z_{i})$ . The $M=\left(\begin{array}{c} m+q-1\\ q \end{array}\right)$ functions $\phi_{i}$ are linearly independent polynomials spanning the space of polynomials in $R^{q}$ of degree less than m .^44^ Furthermore,

$$\begin{array}{r} \eta_{mq}(r)=\left\{ \begin{array}{ll} \frac{(-1{)}^{m+1+q/2}}{2^{2m-1}\pi^{q/2}(m-1)!(m-q/2)!}r^{2m-q}\log\;(r), & \text{ if }\;q\;\text{is even}\\ \frac{\Gamma (q/2-m)}{2^{2m}\pi^{q/2}(m-1)!}r^{2m-q}, & \text{ if }\;q\;\text{is odd}. \end{array}\right. \end{array}$$

The R software allows fitting of thin plate spline using the “gam” function in the package “mgcv.”

The rest of the article is organized as follows. In Section 2, we discuss about the mixture cure rate model framework for interval-censored data and develop an estimation procedure based on the expectation–maximization (EM) algorithm that employs the SVM to model the incidence part. In Section 3, a detailed simulation study is carried out to demonstrate the performance of our proposed model in terms of flexibility, accuracy, and robustness. Comparison of our model with the logistic regression-based and spline regression-based mixture cure rate models in the presence of interval censored data is made in this section. The model performance is further examined and illustrated in Section 4 through the NASA’s Hypobaric Decompression Sickness Database (HDSD). Finally, we end our discussion by some concluding remarks and possible future research directions in Section 5.

## 2. SVM-based mixture cure rate model with interval censoring

### 2.1. Censoring scheme and modeling lifetimes

The data we observe in situations with interval censoring are of the form $\left(L_{i},R_{i},\delta_{i},x_{i},z_{i}\right)$ for i=1,…,n , where n denotes the sample size. For the i -th subject, $L_{i}$ denotes the last inspection time before the event and $R_{i}$ denotes the first subsequent inspection time just after the event. Note that $L_{i}<R_{i}$ . The censoring indicator is denoted by $\delta_{i}=I(R_{i}<\infty )$ , which takes the value 0 if $R_{i}=\infty$ , meaning that the event is not observed for a subject before the last inspection time, and takes the value 1 if $R_{i}<\infty$ , meaning that the event took place but its exact time is not known and is only known to belong to the interval $\left[L_{i},R_{i}\right]$ . To demonstrate the effect of covariates on the latency part, we consider a proportional hazards structure to model the lifetime distribution of the susceptible or non-cured subjects. That is, for the susceptible subjects, we model the hazard function by

(6) $$h_{u}(t_{i}|x_{i})=h_{0}(t_{i})\exp\;\left\{x_{i}^{T}\gamma \right\}$$

where $\gamma =(\gamma_{1},\ldots ,\gamma_{p}{)}^{T}$ is the p -dimensional regression parameter vector measuring the effects of x and $h_{0}(\cdot )$ is an unspecified baseline hazard function. Using (6), we can express (2) as

(7) $$S_{p}(t_{i}|x_{i},z_{i})=1-\pi (z_{i})+\pi (z_{i})\{S_{0}(t_{i}){\}}^{\exp\;(x_{i}^{T}\gamma )}$$

where $S_{0}(\cdot )$ is the baseline survival function (unspecified) corresponding to $h_{0}(\cdot )$ . In this article, we propose to estimate $S_{0}(\cdot )$ using the non-parametric Turnbull estimator,^46^ thereby avoiding any parametric distributional assumption.^47^ Such an estimator does not have any closed form and is developed as an iterative procedure. The steps involved can be described as follows:

a. Using all the $L_{i}$ ’s and $R_{i}$ ’s, i=1,2,…,n , create a grid of time points as $0=\tau_{0}<\tau_{1}<\cdots <\tau_{k}$ .
b. For each i , define a weight $U_{ij}$ that takes the value 1 if the interval $\left(\tau_{j-1},\tau_{j}\right]$ is contained in the interval $\left(L_{i},R_{i}\right]$ , and takes the value 0 otherwise.
c. Make an initial guess of the survival probability at $\tau_{j}$ (say, $S_{0}^{\left(0\right)}(\tau_{j})$ ) for each j .
d. Calculate $p_{j}=S_{0}^{\left(0\right)}(\tau_{j-1})-S_{0}^{\left(0\right)}(\tau_{j})$ , j=1,2,…,k , which denotes the probability of an event to occur at time $\tau_{j}$ .
e. Estimate the number of events that occurred at time $\tau_{j}$ by $e_{j}=\sum_{i=1}^{n}\frac{U_{ij}p_{j}}{\sum_{m}U_{im}p_{m}}$ , where the denominator in $e_{j}$ is the total probability assigned to possible event times in the interval $\left(L_{i},R_{i}\right]$ .
f. Calculate the estimated number of subjects at risk at time $\tau_{j}$ by $Y_{j}=\sum_{l=j}^{k}e_{l}$ .
g. Calculate the updated product-limit estimator of survival function at $\tau_{j}$ using the data $\left(e_{j},Y_{j}\right)$ , say $S_{0}^{\left(1\right)}(\tau_{j})$ , j=1,2,…,k .
h. If $|S_{0}^{\left(1\right)}(\tau_{j})-S_{0}^{\left(0\right)}(\tau_{j})|<\epsilon$ for all j , where ϵ is a tolerance, stop the iterative algorithm. Otherwise, repeat step d through step g using $S_{0}^{\left(1\right)}(\tau_{j})$ .

### 2.2. Form of the likelihood function

As missing observations are inherent to the problem set-up and model framework, we propose to employ the EM algorithm to estimate the unknown parameters.^7,8,48,49^ For implementing the EM algorithm, we need the form of the complete data likelihood function. Let us define $\Delta_{0}=\{i:\delta_{i}=0\}$ and $\Delta_{1}=\{i:\delta_{i}=1\}$ . Missing observations that appear in this context are in terms of the cure indicator variable J , where J is as defined in (1). Note that $J_{i}$ ’s are all known to take the value 1 if $i\in \Delta_{1}$ . However, if $i\in \Delta_{0}$ , $J_{i}$ can either take 0 or 1, and is thus unknown or missing. Using these $J_{i}$ ’s as the missing data, we can define the complete data as $\left(L_{i},R_{i},\delta_{i},J_{i},x_{i},z_{i}\right)$ , for i=1,…,n , which contain both observed and missing data. Under the interval censoring mechanism, we can now express the complete data likelihood function and log-likelihood function as:

(8) $$\begin{array}{rl} L_{c} & =\prod_{i\in \Delta_{1}}{\left[\pi (z_{i})\left\{S_{u}(L_{i}|x_{i})-S_{u}(R_{i}|x_{i})\right\}\right]}^{J_{i}}\\ & \;\times \prod_{i\in \Delta_{0}}(1-\pi (z_{i}){)}^{1-J_{i}}{\left\{\pi (z_{i})S_{u}(L_{i}|x_{i})\right\}}^{J_{i}} \end{array}$$

and

(9) $$\begin{array}{rl} l_{c} & =\sum_{i\in \Delta_{1}}J_{i}\left[\log\;\pi (z_{i})+\log\;\left\{S_{u}(L_{i}|x_{i})-S_{u}(R_{i}|x_{i})\right\}\right]\\ & \;+\sum_{i\in \Delta_{0}}(1-J_{i})\log\;(1-\pi (z_{i}))+J_{i}\left\{\log\;\pi (z_{i})+\log\;S_{u}(L_{i}|x_{i})\right\} \end{array}$$

where $S_{u}(t_{i}|x_{i})=\{S_{0}(t_{i}){\}}^{\exp\;(x_{i}^{T}\gamma )}$ . It can be further noted that

(10) $$l_{c}=l_{c1}+l_{c2}$$

where

(11) $$l_{c1}=\sum_{i=1}^{n}\left[J_{i}\log\;\pi (z_{i})+(1-J_{i})\log\;(1-\pi (z_{i}))\right]$$

is a function that depends on the incidence part only and

(12) $$l_{c2}=\sum_{i=1}^{n}\left[\delta_{i}\log\;\left\{S_{u}(L_{i}|x_{i})-S_{u}(R_{i}|x_{i})\right\}+(1-\delta_{i})J_{i}\log\;S_{u}(L_{i}|x_{i})\right]$$

is a function that depends on the latency part only.

### 2.3. Modeling the incidence part with SVM

Let us assume that $J_{i}$ for $i\in \Delta_{0}$ are observed by some mechanism to assist our theory. SVM algorithm maximizes the linear or nonlinear margin between the two closest points belonging to the opposite classification groups (cured and susceptible). That is, SVM solves the following optimization problem for $d_{i};i=1,\ldots ,n$ :

(13) $$\max_{d_{1},\ldots ,d_{n}}\left[-\frac{1}{2}\sum_{i=1}^{n}\sum_{j=1}^{n}d_{i}d_{j}(2J_{i}-1)(2J_{j}-1)\Phi_{k}(z_{i},z_{j})+\sum_{i=1}^{n}d_{i}\right]$$

subject to the constraint $\sum_{i=1}^{n}(2J_{i}-1)d_{i}=0$ and $0\leq d_{i}\leq C$ , for i=1,…,n , where C is a parameter that trades off between the margin width and misclassification proportion. Smaller values of C cause optimizer to look for a larger margin width allowing higher misclassification. $\Phi_{k}(.,.)$ is a symmetric positive semi-definite kernel function, which we consider to be the radial basis function (RBF) given by $\Phi_{k}(z_{i},z_{j})=\exp\;\left\{-\frac{\left(z_{i}-z_{j}{)}^{T}(z_{i}-z_{j}\right)}{2\sigma^{2}}\right\}$ . RBF is a popular choice of the kernel function owing to its robustness by implementing the idea that a linear classifier in higher dimension can be used as a nonlinear classifier in lower dimension. The parameter $\sigma^{2}$ determines the kernel-width. Both hyper-parameters C and $\sigma^{2}$ are to be tuned to obtain the highest classification accuracy using cross-validation methods.^50^ Grid search can be implemented to determine C and $\sigma^{2}$ . Low values of $\sigma^{2}$ result in overfitting and jagged separator, while high values of $\sigma^{2}$ result in more linear and smoother decision boundaries. Also, it is recommended to standardize the covariate vector z .

The mapping $J_{i}$ to $2J_{i}-1$ converts the respective 0 and 1s to −1 and + 1s, which aids in formulation of the optimization problem under the SVM framework. Once $d_{i}$ ’s are obtained, we can derive a threshold b as $b=\sum_{i=1}^{n}(2J_{i}-1)d_{i}\Phi_{k}(z_{i},z_{j})-(2J_{j}-1)$ , for some $d_{j}>0$ . For any new covariate vector $z_{new}$ , the optimal decision or classification rule is given by

(14) $$\psi (z_{new})=\sum_{i=1}^{n}d_{i}(2J_{i}-1)\Phi_{k}(z_{i},z_{new})-b.$$

As suggested by Li et al.,^31^ the sequential minimal optimization (SMO) method^51^ can be applied to solve (13). As opposed to solving large quadratic optimization problems to train an SVM model, SMO solves a series of smallest possible quadratic problems. Thus, SMO is relatively time inexpensive algorithm. Any subject with covariate $z_{new}$ is assigned to the susceptible group if $\psi (z_{new})>0$ and to the cured group if $\psi (z_{new})<0$ .

In the given context, note that it is not enough to just classify subjects as being cured or susceptible. It is also of our interest to obtain the estimates of uncured probabilities $\pi (z_{i})$ or equivalently the cured probabilities $1-\pi (z_{i})$ . For this purpose, we use the Platt scaling method to obtain an estimate of $\pi (z_{i})$ from the classification rule ψ(.) .^52^ The estimate of $\pi (z_{i})$ by Platt scaling method is given by

(15) $$\hat{\pi }(z_{i})=\frac{1}{1+\exp\;\{A\psi (z_{i})+B\}}$$

where A and B are obtained by maximizing the following function:

(16) $$\sum_{i=1}^{n}(1-\zeta_{i})[A\psi (z_{i})+B]-\log\;[1+\exp\;\{A\psi (z_{i})+B\}].$$

Here

(17) $$\zeta_{i}=\left\{ \begin{array}{ll} \frac{n^{\left(1\right)}+1}{n^{\left(1\right)}+2}, & \text{ if }J_{i}=1\\ \frac{1}{n^{\left(0\right)}+2}, & \text{ if }J_{i}=0 \end{array}\right.$$

and $n^{\left(1\right)}$ and $n^{\left(0\right)}$ represents the number of subjects in the susceptible and cured groups, respectively.

We started our discussion on the SVM-based modeling of the incidence part above with the assumption that $J_{i}$ s are observed and available for training purpose. However, in practice, the cure status $J_{i}$ is not known for $i\in \Delta_{0}$ . Multiple imputation-based approach can be applied here to obtain $\hat{\pi }(z_{i})$ with imputed values of $J_{i}$ for i=1,…,n . Note that the proposed multiple imputation technique does not rely on naive assumptions such as the existence of a known threshold time beyond which all censored observations can be considered cured.^53^ The steps of multiple imputation are as follows:

1. For a pre-defined integer $N^{*}$ and $n^{*}=1,2,\ldots ,N^{*}$ , generate $\left\{J_{i}^{\left(n^{*}\right)}:i=1,\ldots ,n\right\}$ , where $J_{i}^{\left(n^{*}\right)}$ is a Bernoulli random variable with success probability $p_{i}^{\left(n^{*}\right)}$ . The discussion on deriving $p_{i}^{\left(n^{*}\right)}$ is provided in Section 2.5.
2. For the imputed data $\left\{J_{i}^{\left(n^{*}\right)}:i=1,\ldots ,n\right\}$ , obtain ${\hat{\pi }}^{\left(n^{*}\right)}(z_{i})$ as the estimate of $\pi (z_{i})$ by employing the SVM followed by the Platt scaling method given in (15) for $n^{*}=1,2,\ldots ,N^{*}$ .
3. Calculate $\hat{\pi }(z_{i})=(1/N^{*})\sum_{n^{*}=1}^{N^{*}}{\hat{\pi }}^{\left(n^{*}\right)}(z_{i})$ as the final estimate of $\pi (z_{i})$ .

### 2.4. Tuning the SVM model

To address the issue with over/under fitting, we split the data into two sets, namely, the training set and the testing set. The training set is used to obtain the optimal hyper-parameters of the SVM model and then those optimal hyper-parameters are used to train the optimal SVM model. On the other hand, the testing set is used to test or validate the final SVM model. We examine two most critical hyper-parameters of the SVM, namely, C and σ when training the SVM model. The parameter C is a regularization parameter ( $l_{2}$ ) that penalizes the model for any mis-classification. The value of C is inversely proportional to the strength of the regularization. When the value of the C is large, the penalty for mis-classification is substantial, and the strength of the regularization is small and vice versa. The parameter σ of the RBF kernel on the other hand controls the similarity of the impact of a single training point, which influences the performance of the model. These hyper-parameters can be obtained using cross-validation techniques.^54,50^ In this article, we use the grid search cross-validation technique to obtain the optimal hyper-parameters of the SVM model. For the grid search cross-validation technique, we fit several pairwise models using different sets of hyper-parameter values. These fitted models are then evaluated to obtain the best optimal trained model and hyper-parameter values. These optimal hyper-parameter values from the optimal selected model are then used to fit the final model. Finally, we validate the performance of the final fitted SVM model by performing predictions using the testing set. Model performance evaluation criteria such as the graphical receiver operating characteristic (ROC) curve and it’s area under the curve (AUC) are used to evaluate the performance of the final model.

### 2.5. Development of the EM algorithm

The E-step in the EM algorithm involves finding the conditional expectation of the complete data log-likelihood function in (9) given the current estimates (say, at the (r+1) -th iteration step) and the observed data, which is equivalent to finding the conditional expectation of $J_{i}$ given the observed data, $\pi (z_{i})$ and $(S_{0}(\cdot ),\gamma^{T}{)}^{T}$ , as

(18) $$w_{i}^{\left(r+1\right)}=\delta_{i}+(1-\delta_{i})\frac{\pi^{\left(r\right)}(z_{i})S_{u}^{\left(r\right)}(L_{i}|x_{i})}{1-\pi^{\left(r\right)}(z_{i})+\pi^{\left(r\right)}(z_{i})S_{u}^{\left(r\right)}(L_{i}|x_{i})},\;\;i=1,\ldots ,n$$

where $S_{u}^{\left(r\right)}(L_{i}|x_{i})=\{{\hat{S}}_{0}(L_{i}){\}}^{\exp\;(x_{i}^{T}\gamma^{\left(r\right)})}$ with ${\hat{S}}_{0}(L_{i})$ denoting a non-parametric estimator of the baseline survival function evaluated at $L_{i}$ , i=1,2,…,n . Note that (18) implies that $w_{i}^{\left(r+1\right)}=1$ for all $i\in \Delta_{1}$ . We obtain the conditional expectation of $l_{c}$ by simply replacing $J_{i}$ ’s with $w_{i}^{\left(r+1\right)}$ in (9). We denote the aforementioned conditional expectation by

(19) $$Q_{c}=Q_{c1}+Q_{c2}$$

where

(20) $$Q_{c1}=\sum_{i=1}^{n}\left[w_{i}^{\left(r+1\right)}\log\;\pi (z_{i})+(1-w_{i}^{\left(r+1\right)})\log\;(1-\pi (z_{i}))\right]$$

and

(21) $$Q_{c2}=\sum_{i=1}^{n}\left[\delta_{i}\log\;\left\{S_{u}(L_{i}|x_{i})-S_{u}(R_{i}|x_{i})\right\}+(1-\delta_{i})w_{i}^{\left(r+1\right)}\log\;S_{u}(L_{i}|x_{i})\right].$$

The M-step updates the parameters in $Q_{c1}$ and $Q_{c2}$ . For r=0,1,… , the procedure for the (r+1) -th iteration step of the EM algorithm is given below.

1. Carry out the multiple imputation technique, as described in Section 2.3, by considering $p_{i}^{\left(n^{*}\right)}=w_{i}^{\left(r+1\right)}$ , for $n^{*}=1,\ldots ,N^{*}$ and i=1,…,n . Obtain ${\hat{\pi }}^{\left(r+1\right)}(z_{i})=(1/N^{*})\sum_{n^{*}=1}^{N^{*}}{\hat{\pi }}^{\left(n^{*}\right)}(z_{i})$ by applying the Platt scaling method with the classification rule ψ(⋅) defined in (14). Recall that the classification rule is built based on the imputed data $\left\{J_{i}^{\left(n^{*}\right)}:i=1,\ldots ,n\right\}$ , where $J_{i}^{\left(n^{*}\right)}$ is a Bernoulli random variable with success probability $p_{i}^{\left(n^{*}\right)}$ .
2. Obtain $\gamma^{\left(r+1\right)}$ by maximizing the function $Q_{c2}$ , as defined in (21), with respect to γ . That is, find
(22) $$\gamma^{\left(r+1\right)}=\underset{\gamma }{\arg\;\max}\;Q_{c2}.$$
  The maximization in (22) can be carried out by using the “optim()” function in R software and by specifying the method as “Nelder-Mead.” In this regard, one may also look at new optimization methods based on nonlinear conjugate gradient algorithm with an efficient line search technique.^55–57^
3. Check for the convergence as follows:
$$\begin{array}{r} ||\theta^{\left(r+1\right)}-\theta^{\left(r\right)}|{|}_{2}^{2}<\epsilon \end{array}$$
  where $\theta^{\left(k\right)}=(\bar{\pi^{\left(k\right)}}(z),\gamma^{(k)T}{)}^{T}$ , with $\bar{\pi^{\left(k\right)}}(z)=\frac{1}{n}\sum_{i=1}^{n}\pi^{\left(k\right)}(z_{i})$ , ϵ>0 is some pre-determined and sufficiently small tolerance and $||\cdot |{|}_{2}$ is the $L_{2}$ -norm. If the above criterion is satisfied, then, stop the algorithm. In this case, ${\hat{\pi }}^{\left(r+1\right)}(z_{i})$ , for i=1,…,n , and $\gamma^{\left(r+1\right)}$ are the final pointwise estimates. On the other hand, if the above criterion is not met, continue to Step 4.
4. Update $w_{i}^{\left(r+1\right)}$ in (18) to
(23) $$w_{i}^{\left(r+2\right)}=\delta_{i}+(1-\delta_{i})\frac{{\hat{\pi }}^{\left(r+1\right)}(z_{i})S_{u}^{\left(r+1\right)}(L_{i}|x_{i})}{1-{\hat{\pi }}^{\left(r+1\right)}(z_{i})+{\hat{\pi }}^{\left(r+1\right)}(z_{i})S_{u}^{\left(r+1\right)}(L_{i}|x_{i})}.$$
5. Repeat steps 1 to 4 until convergence is achieved.

Note that maximization of $Q_{c2}$ with respect to γ can be done only after estimating the baseline survival function $S_{0}(\cdot )$ which appears as a nuisance parameter in (21). As mentioned in Section 2.1, we estimate $S_{0}(\cdot )$ using the non-parametric Turnbull estimator.

### 2.6. Calculating the standard errors

The standard errors are estimated by non-parametric bootstrapping. For $b^{\prime }=1,\ldots ,B$ , $b^{\prime }$ -th bootstrapped data set is obtained by resampling with replacement from the original data. The sample size of the $b^{\prime }$ -th bootstrapped data is the same as the original data. Then, we carry out steps 1 to 5 of the EM algorithm as detailed in Section 2.5 to obtain the estimates of model parameters for each bootstrapped data. This gives us B estimates for each model parameter. For each parameter, the standard deviation of these B estimates provides an estimate of the standard error of the parameter.

### 2.7. Initial values of model parameters

To start the iterative EM algorithm, we need to come up with initial values of $\pi (z_{i})$ , for i=1,…,n , and γ . To provide an initial guess of $\pi (z_{i})$ , we can consider the censoring indicator $\delta_{i}$ as the cure indicator. That is, we consider $J_{i}=0$ if $\delta_{i}=0$ and $J_{i}=1$ if $\delta_{i}=1$ for i=1,2,…,n . Then, we employ the SVM to come up with the classification rule (as in (14)) and finally, apply the Platt scaling method (as in (15)) to obtain $\pi (z_{i})$ . On the other hand, to provide an initial guess for the latency parameter γ , we can simply initiate each component in γ by 0.5.

**Figure 1.**
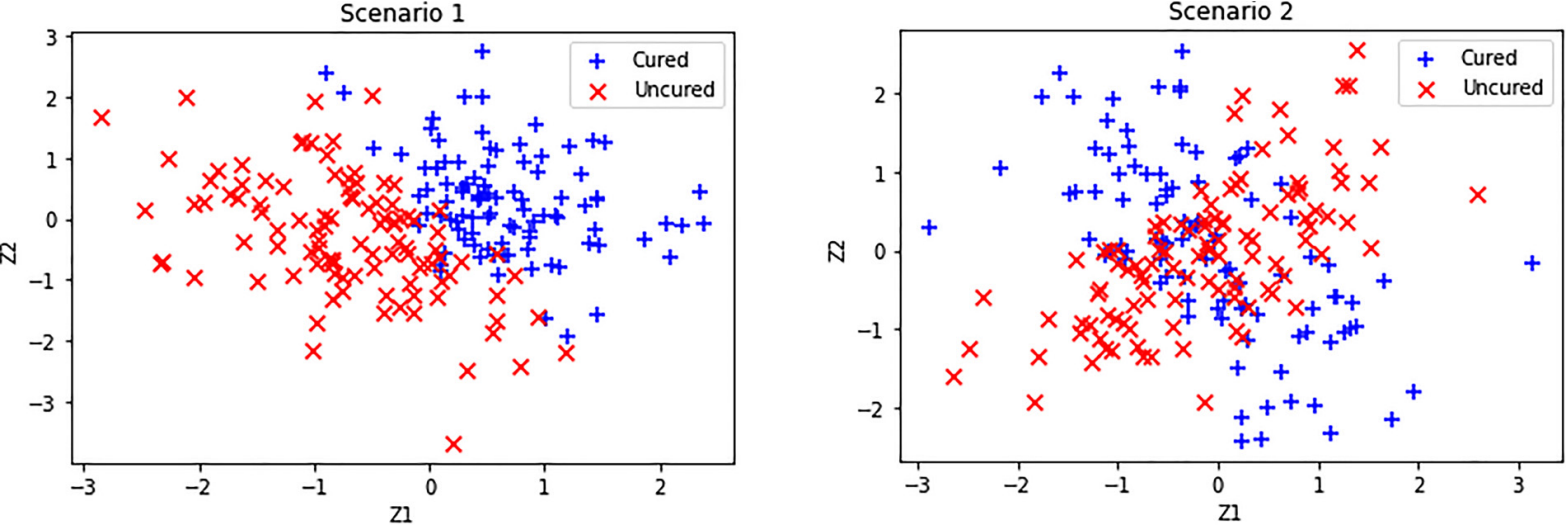
Simulated cured and uncured observations for Scenarios 1 and 2 considered.

**Figure 2.**
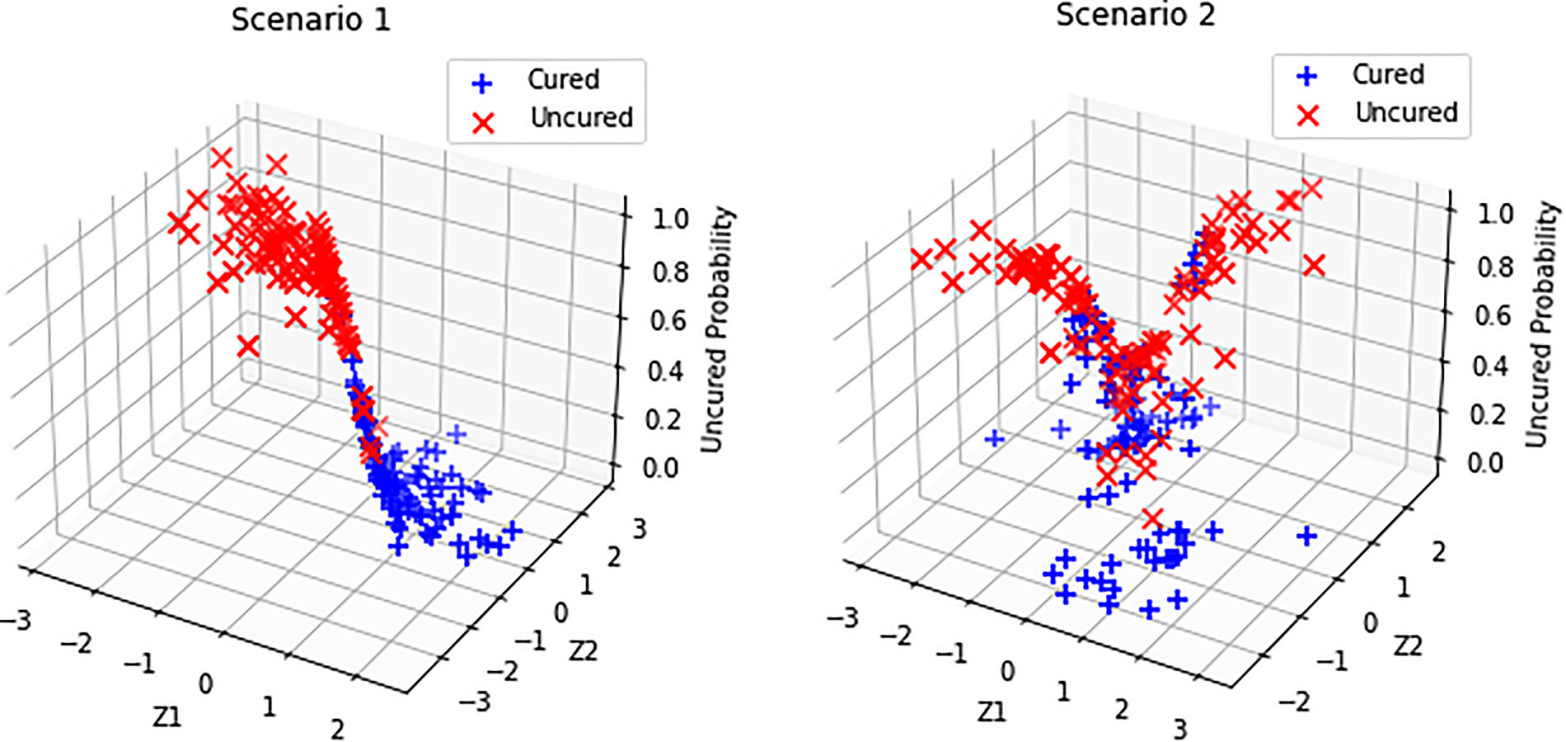
Simulated uncured probabilities and their behavior with respect to the covariates for Scenarios 1 and 2.

## 3. Simulation study

In this section, we assess the performance of the proposed SVM-based EM algorithm to estimate the model parameters of the mixture cure rate model for interval censored data. We also compare the performance of the SVM-based EM algorithm with the logistic regression-based and spline regression-based EM algorithms. To fit the thin plate spline for the incidence part, we use the “gam” function in the package “mgcv.” We consider the following scenarios using which we generate the true uncured probabilities π(z) :

$$\begin{array}{rl} \text{Scenario 1:}\;\pi (z) & =\frac{e^{0.3-5z_{1}-3z_{2}}}{1+e^{0.3-5z_{1}-3z_{2}}}\\ \text{Scenario 2:}\;\pi (z) & =\frac{e^{0.3+10z_{1}z_{2}-5z_{1}z_{2}}}{1+e^{0.3+10z_{1}z_{2}-5z_{1}z_{2}}}\\ \text{Scenario 3:}\;\pi (z) & =\exp\;(-\exp\;(-0.8z_{1}z_{2}+1.1z_{2}z_{4}+0.5z_{3}+0.2z_{7}^{2}-1.3\text{sin}(z_{5}z_{6})\\ & \;+1.9\text{cos}(z_{7}z_{8})-1.5\exp\;(z_{5}z_{6}z_{7})-1.6z_{7}z_{8}z_{9}z_{10}+0.8z_{6}z_{7}z_{8}^{2}z_{9}^{2}\\ & \;+1.8\text{cos}(z_{5}z_{6}z_{7}z_{8}z_{9})+1.2|(z_{6}z_{7}z_{8}z_{9}z_{10}){|}^{0.5}-2.4)). \end{array}$$

In Scenarios 1 and 2, $z_{1}$ and $z_{2}$ are generated from the standard normal distribution. In Scenario 3, $z_{1},z_{2},z_{3}$ , and $z_{4}$ are generated from the Bernoulli distribution with success probabilities 0.5, 0.3, 0.5, and 0.7, respectively, whereas $z_{5},z_{6},\ldots ,z_{10}$ are generated from the standard normal distribution. In all scenarios, we consider z=x ,that is, we use the same set of covariates to model the incidence and latency parts. Note that Scenario 1 represents the standard logistic regression model which captures a linear classification boundary, whereas Scenario 2 captures nonlinear classification boundary (see Figure 1). On the other hand, Scenario 3 represents a more complex link function with a large number of covariates and complicated interaction terms. Corresponding to Scenarios 1 and 2, Figure 2 shows the plots of simulated uncured probabilities and how they vary with respect to the covariates $z_{1}$ and $z_{2}$ . We consider different sample sizes as n=300 and n=600 . We assume lifetimes of the susceptible subjects follow the proportional hazards structure with the hazard function

$$\begin{array}{r} h_{u}(t)=\alpha t^{\alpha -1}\exp\;\left\{x^{T}\gamma \right\} \end{array}$$

where the true value of α is chosen as 1 for Scenarios 1 and 2, and 3.5 for Scenario 3. For Scenarios 1 and 2, we consider the true value of γ as (−5,10) , whereas for Scenario 3 we consider γ=(−0.8,1.5,0.5,1.3,−0.6,−1.4,−0.5,−0.8,0.5,1.8) . The censoring time is generated from a uniform distribution in (0,20) . Under these settings, the true cure probability and censoring proportion, denoted by (cure, censoring), for Scenarios 1, 2, and 3 are roughly (0.50, 0.65), (0.40, 0.60), and (0.60, 0.70), respectively. Thus, using the three scenarios we cover low, moderate, and high cure and censoring rates. To generate interval censored lifetime data $(L_{i},R_{i},\delta_{i}),i=1,2,\ldots ,n$ , we carry out the following steps:

Step 1: Generate a Uniform (0,1) random variable $U_{i}$ and a censoring time $C_{i}$ ;
Step 2: If $U_{i}\leq 1-\pi (z_{i}),$ set $T_{i}=\infty$ ;
Step 3: If $U_{i}>1-\pi (z_{i}),$ generate $T_{i}$ from a Weibull distribution with shape parameter α and scale parameter $\{\exp\;(\gamma_{1}x_{1i}+\gamma_{2}x_{2i}){\}}^{-\frac{1}{\alpha }}$ ;
Step 4:
  a. If $\min\{T_{i},C_{i}\}=C_{i}$ , set $L_{i}=C_{i}$ , $R_{i}=\infty$ , and $\delta_{i}=0$ ;
  b. If $\min\{T_{i},C_{i}\}=T_{i}$ , set $\delta_{i}=1$ , and generate $L_{1i}$ from Uniform (0.2,0.7) distribution and $L_{2i}$ from Uniform (0,1) distribution. Next, create intervals $(0,L_{2i}],(L_{2i},L_{2i}+L_{1i}],\ldots ,(L_{2i}+k\times L_{1i},\infty ],k=1,2,\ldots ,$ and select $\left(L_{i},R_{i}\right)$ that satisfies $L_{i}<T_{i}\leq R_{i}$ .

**Table 1.** Comparison of bias of the uncured probability and susceptible survival probability for different models.

		Uncured probability			Susceptible survival probability		
		Bias			Bias		
n	Scenario	SVM	Spline	Logistic	SVM	Spline	Logistic
300	1	−0.1425	−0.1632	0.0584	0.1079	0.1101	0.1060
	2	−0.0684	0.0900	0.2322	0.0500	0.0505	0.0515
	3	0.0544	0.1046	0.1786	0.1058	0.0651	0.1013
600	1	−0.1255	−0.1611	0.0474	0.1075	0.1089	0.1058
	2	−0.0628	0.1009	0.2186	0.0492	0.0495	0.0511
	3	0.0364	0.0957	0.1494	0.0828	0.0774	0.1034

SVM: support vector machine.

All simulations are done using the R statistical software (Version 4.0.4) and results are based on M=500 Monte Carlo runs. The computational codes for data generation and SVM-based EM algorithm are available in the Supplemental Material. In all cases, 67% of the data is used as training set and the remaining 33% of the data is used as testing set. To employ our proposed methodology, we consider the number of imputations in the multiple imputation technique to be 5, which is in line with existing works.^31,58^ In Tables 1 and 2, we report the bias and mean squared error (MSE), respectively, of the estimated uncured probability $\hat{\pi }(z)$ and the susceptible survival probability $\hat{S_{u}}=\hat{S_{u}}(.,.;x)$ . These are calculated as:

$$\begin{array}{rl} \text{Bias}(\hat{\pi }(z)) & =\frac{1}{M}\sum_{k=1}^{M}[\frac{1}{n}\sum_{i=1}^{n}\{\hat{\pi^{\left(k\right)}}(z_{i})-\pi^{\left(k\right)}(z_{i})\}]\\ \text{Bias}(\hat{S_{u}}) & =\frac{1}{M}\sum_{k=1}^{M}[\frac{1}{n}\sum_{i=1}^{n}\{\hat{S_{u}^{\left(k\right)}}(L_{i},R_{i};x_{i})-S_{u}^{\left(k\right)}(L_{i},R_{i};x_{i})\}]\\ \text{MSE}(\hat{\pi }(z)) & =\frac{1}{M}\sum_{k=1}^{M}[\frac{1}{n}\sum_{i=1}^{n}\{\hat{\pi^{\left(k\right)}}(z_{i})-\pi^{\left(k\right)}(z_{i}){\}}^{2}]\\ \text{MSE}(\hat{S_{u}}) & =\frac{1}{M}\sum_{k=1}^{M}[\frac{1}{n}\sum_{i=1}^{n}\{\hat{S_{u}^{\left(k\right)}}(L_{i},R_{i};x_{i})-S_{u}^{\left(k\right)}(L_{i},R_{i};x_{i}){\}}^{2}] \end{array}$$

where $\pi^{\left(k\right)}(z_{i})$ and $S_{u}^{\left(k\right)}(L_{i},R_{i};x_{i})$ are the true uncured probability and susceptible survival probability, respectively, corresponding to the i -th subject and the k -th Monte Carlo run. Similarly, $\hat{\pi^{\left(k\right)}}(z_{i})$ and $\hat{S_{u}^{\left(k\right)}}(L_{i},R_{i};x_{i})$ are the estimated uncured probability and susceptible survival probability, respectively, corresponding to the i -th subject and the k -th Monte Carlo run. In the above expressions, note that $S_{u}^{\left(k\right)}(L_{i},R_{i};x_{i})=S_{u}^{\left(k\right)}(T_{i};x_{i}),$ where $T_{i}=\frac{L_{i}+R_{i}}{2}$ if $R_{i}<\infty$ and $T_{i}=L_{i}$ if $R_{i}=\infty$ . $\hat{S_{u}^{\left(k\right)}}(L_{i},R_{i};x_{i})$ is defined in a similar way.

**Table 2.** Comparison of MSE of the uncured probability and susceptible survival probability for different models.

		Uncured probability			Susceptible survival probability		
		MSE			MSE		
n	Scenario	SVM	Spline	Logistic	SVM	Spline	Logistic
300	1	0.1132	0.1753	0.0618	0.1019	0.1085	0.1022
	2	0.0827	0.1906	0.2184	0.0338	0.0363	0.0598
	3	0.1052	0.1587	0.2111	0.0609	0.0793	0.1060
600	1	0.1128	0.1715	0.0614	0.0988	0.1001	0.1020
	2	0.0809	0.1901	0.2185	0.0328	0.0340	0.0727
	3	0.0956	0.1280	0.1696	0.0380	0.0468	0.0649

SVM: support vector machine; MSE: mean squared error.

From Table 1, it is clear that the biases of the estimated uncured probability and the susceptible survival probability obtained from the logistic regression-based EM algorithm is smaller than those obtained from the proposed SVM-based EM algorithm as well as the spline-based EM algorithm when logistic regression is the correct model (Scenario 1). However, when the true model for the uncured probability is not the logistic regression in Scenarios 2 and 3, the proposed SVM-based EM algorithm produces smaller bias in the estimated uncured probability when compared to both logistic regression-based and spline-based EM algorithms. In this case, as far as the estimated susceptible survival probabilities are concerned, the SVM-based EM algorithm produces smaller bias only under Scenario 2. From Table 2, we note that when the logistic regression is the true model for the uncured probability (i.e. under Scenario 1), the MSE of the estimated uncured probability obtained from the logistic regression-based EM algorithm is smaller than those obtained from the SVM-based and spline-based EM algorithms. However, under Scenarios 2 and 3, that is, under non-logistic true models for the uncured probability, the proposed SVM-based EM algorithm produces smaller MSE of the estimated uncured probability when compared to both logistic regression-based and spline-based EM algorithms. When it comes to the estimation of the susceptible survival probability, our proposed SVM-based EM algorithm produces smaller MSEs in all considered scenarios. Overall, we can conclude that the proposed SVM-based EM algorithm performs better than the standard logistic regression-based and spline-based EM algorithms when the true classification boundary is nonlinear and complex. This clearly demonstrates the ability of the proposed SVM-based mixture cure model to handle complex nonlinear classification boundaries.

**Figure 3.**
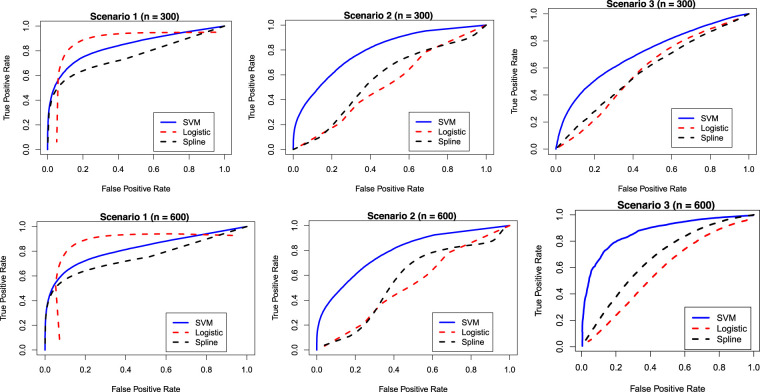
Receiver operating characteristic (ROC) curves for different models and under different scenarios.

Although, in practice, the cured status is unobserved for a real data, we do know which observations can be considered as cured when we simulate data. Using such information on the cured status for simulated data, we can easily compare the proposed SVM-based mixture cure model with the logistic regression-based and spline regression-based mixture cure models using the ROC curves and the AUC values for different scenarios we have considered. Note that the true label for calculating the AUC is the true cure index for each subject when the data is generated. Figure 3 presents the ROC curves under different scenarios. The corresponding AUC values are presented in Table 3. These results are based on 500 Monte Carlo runs. It is once again clear that under Scenarios 2 and 3 (i.e. when the true classification boundaries are nonlinear), the performance (or the accuracy) of the SVM-based mixture cure model is much better than the logistic regression-based and the spline-based mixture cure models. However, under Scenario 1 (i.e. when the true classification boundary is linear), the logistic regression-based model performs slightly better than the SVM-based model. The similarity in the AUC values obtained from the training data and testing data implies that there is no issue with over/under fitting.

**Table 3.** Comparison of AUC values for different models and scenarios.

		Training AUC			Testing AUC		
*n*	Scenario	SVM	Spline	Logistic	SVM	Spline	Logistic
300	1	0.8476	0.7461	0.9248	0.8437	0.7409	0.9225
	2	0.8057	0.5756	0.5330	0.7990	0.5562	0.5445
	3	0.8831	0.6964	0.5885	0.7312	0.5837	0.5507
600	1	0.8229	0.7421	0.9227	0.8218	0.7398	0.9215
	2	0.7973	0.5659	0.5255	0.7956	0.5554	0.5432
	3	0.9231	0.6721	0.5812	0.8706	0.6398	0.5615

SVM: support vector machine; AUC: area under the curve.

To further assess the robustness and generalizability of the proposed SVM model across different data settings, we study a scenario where we generate 10 correlated covariates from a multivariate normal distribution, $N_{10}(0,\Sigma )$ , where Σ denotes the variance–covariance matrix whose (i,j) th element, denoted by $\sigma_{ij}$ , is defined as $\sigma_{ij}=0.9^{|i-j|}$ , 1≤i,j≤10 . The choice of 0.9 as the base for exponentiation determines how quickly the correlation increases with decreasing separation. In this scenario, we consider all 10 covariates for the incidence part but only choose a subset of five covariates for the latency part. In this way, we ensure z≠x . The PH model is fitted for the latency with the true value of γ as γ=(−0.8,1.5,0.5,1.3,−0.6) . Table 4 presents the biases and MSEs of the uncured and susceptible survival probabilities, whereas Table 5 presents the AUC values for both training and testing sets. From Tables 4 and 5, it is clear that the SVM once again outperforms both spline and logistic models, thereby demonstrating robustness and generalizability.

**Table 4.** Comparison of different models through the biases and MSEs of different quantities of interest for n=300 and in the presence of correlated covariates, where z≠x .

	Uncured probability		Susceptible survival probability	
Model	Bias	MSE	Bias	MSE
SVM	0.0611	0.0936	0.0966	0.0779
Logistic	0.1740	0.2335	0.0972	0.0936
Spline	0.0949	0.1433	0.0955	0.0825

SVM: support vector machine; MSE: mean squared error.

**Table 5.** Comparison of different models through the AUC values for n=300 and in the presence of correlated covariates, where z≠x .

Model	Training AUC	Testing AUC
SVM	0.8024	0.7462
Logistic	0.5340	0.5431
Spline	0.6497	0.6087

SVM: support vector machine; AUC: area under the curve.

**Table 6.** Comparison of SVM and NN models through the biases and MSEs of different quantities of interest for n=300 .

		Uncured probability		Susceptible survival probability	
Scenario	Model	Bias	MSE	Bias	MSE
1	SVM	−0.0954	0.1395	0.1075	0.1092
	NN	−0.2231	0.2265	0.1074	0.1108
2	SVM	−0.0570	0.0877	0.0494	0.0338
	NN	−0.1846	0.2120	0.0492	0.0352
3	SVM	0.0698	0.1095	0.1172	0.0678
	NN	−0.0990	0.1544	−0.0385	0.0579

SVM: support vector machine; MSE: mean squared error; NN: neural network.

As per the suggestion of a reviewer, we also used the NN to model the incidence part, that is, π(z) , and then the EM algorithm to estimate all parameters. For this purpose, we fitted a two hidden layers NN with (12, 24) number of neurons respectively in the first and second layers. The sigmoid activation function was used to fit the fully connected NN. In Table 6, we present the biases and MSEs of the uncured and susceptible survival probabilities. Clearly, the performance of SVM is better in estimating the uncured probability, which is our main parameter of interest. Regarding the estimation of the susceptible survival probability, the performances are comparable. In Table 7, we compare the AUCs and computation times of SVM and NN models. The computation times represent the time (in seconds) to produce the incidence and latency estimates along with the standard errors (obtained using a bootstrap sample of size 100) for a generated data of size 300. For other sample sizes, the observations are similar and hence not reported for the sake of brevity. Observe that the computing times of the SVM model is much lower than that of the NN model for all three scenarios. Again, the SVM results in higher AUC values, meaning an improved predictive accuracy. These findings allow us to conclude that the proposed SVM model is preferred to the NN model.

**Table 7.** Comparison of SVM and NN models through the AUC values and computation times for n=300 .

Scenario	Model	Training AUC	Testing AUC	Computation time (in seconds)
1	SVM	0.8273	0.8150	86.13
	NN	0.7521	0.7393	111.07
2	SVM	0.7922	0.7675	121.88
	NN	0.7801	0.7094	143.27
3	SVM	0.8791	0.7216	192.35
	NN	0.8558	0.6952	239.16

SVM: support vector machine; AUC: area under the curve; NN: neural network.

## 4. Illustrative example: Analysis of HDSD data

**Figure 4.**
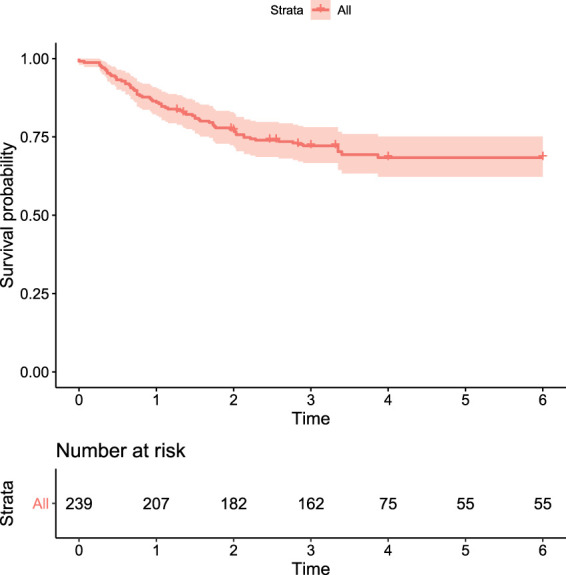
Plot of the NPMLE of the survival function for the HDSD data.

HDSD: Hypobaric decompression sickness database; NPMLE: non-parametric maximum likelihood estimate.

We further demonstrate our proposed methodology using a data set that is extracted from the NASA’s Hypobaric Decompression Sickness Data Bank, hereafter referred as HDSD data.^59^ The data set has information on subjects who underwent denitrogenation test procedures before being exposed to a hypobaric environment. The event of interest is the onset of grade IV venous gas emboli (VGE). The time to onset of grade IV VGE, if it occurred, was not exactly observed but was contained within a time interval. The covariates of interest are age (in years), sex (1: male; 0: female), TR360 which is a measure of decompression stress that ranges from 1.04 to 1.89, and noadyn which is an indicator of whether the subject was ambulatory (noadyn = 1) or lower body adynamic (noadyn = 0) during the test session. Information on 236 subjects is available for downstream analysis whose event times are either interval censored or right censored.^41^ In Figure 4, we present a plot of the non-parametric maximum likelihood estimate (NPMLE) of the survival function. Clearly, we can see that the plot levels off to a significant non-zero proportion. This indicates that there could be a greater likelihood of the presence of cured fraction in the data.

**Figure 5.**
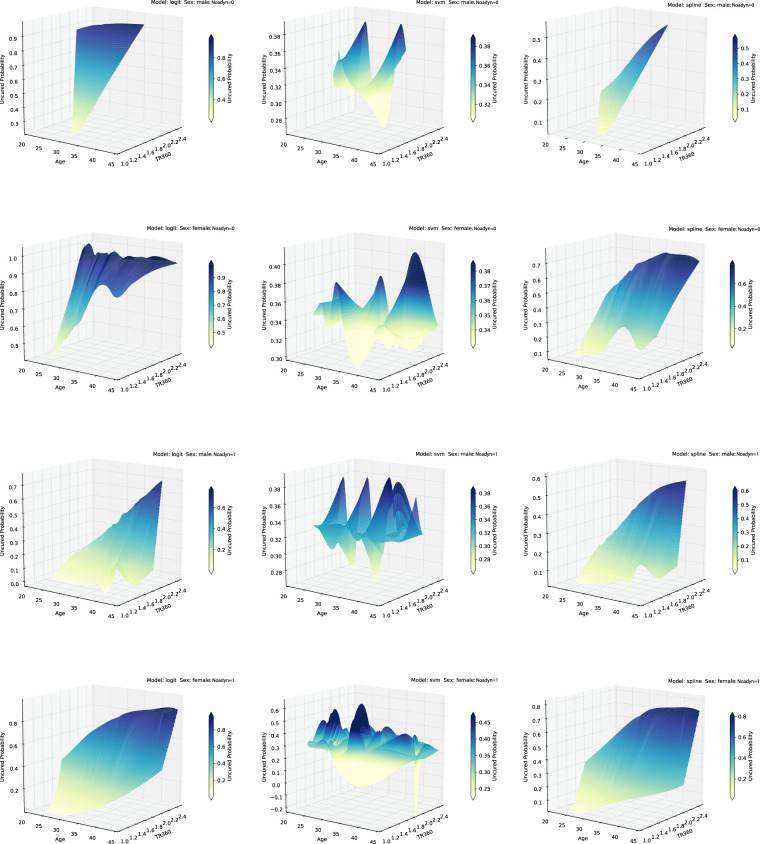
Estimates of uncured probabilities as a function of age and TR360 when stratified by noadyn and sex for the Hypobaric decompression sickness database (HDSD) data.

We fit the proposed SVM-based mixture cure model and, for comparison, we also fit the logistic regression-based and spline regression-based mixture cure models. Noting that the sample size for the HDSD data is small, and to avoid over-fitting or under-fitting, we adopt a 10-fold cross-validation technique that allows us to simultaneously fit and evaluate each model on the full data. This is consistent with Hastie et al.^54^ First, we draw inference on the incidence part of the model. In Figure 5, we plot the estimates of the uncured probabilities against age and TR360 when stratified by sex and noadyn for all models. Clearly, under the proposed SVM-based model, the change in the estimates of the uncured probabilities is non-monotonic with respect to age and TR360. This non-monotonic relationship is not captured by the logistic regression-based and spline regression-based models. Note that for the spline regression-based model, the pattern is similar to the logistic regression-based model.

**Figure 6.**
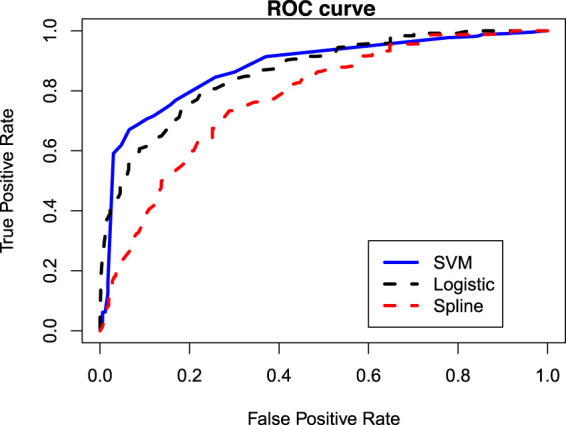
ROC curves under different models for the HDSD data.

HDSD: Hypobaric decompression sickness database; ROC: receiver operating characteristic.

Next, we verify whether our proposed model’s ability to capture nonlinear pattern in the data can result in improved predictive accuracy when predicting the cured/uncured statuses are of interest. This can be verified using the ROC curves and by comparing the AUC values for different models that we have considered. Noting that the cured statuses are unknown for the set of right censored observations in a real data, we first impute the missing cured/uncured statuses. For each right censored observation, the missing uncured status can be imputed by generating a random number from a Bernoulli distribution whose success probability is the conditional probability of uncured, as given in (18). With the complete knowledge of cured/uncured statuses for all subjects, the ROC curves can be drawn and the AUC values can be computed. However, since this method involves simulation (i.e. randomness), we make the ROC curves and the AUC values more consistent by repeating the procedure 500 times and reporting the averaged ROC curves and the averaged AUC values. Figure 6 presents the averaged ROC curves for different models and the corresponding AUC values turn out to be 0.8795, 0.8627, and 0.7766 for the SVM-based, logistic regression-based, and spline regression-based models, respectively. Thus, the proposed SVM-based model indeed provides the highest predictive accuracy for the considered HDSD data.

Finally, we look at the results related to the latency parts of the fitted models. Table 8 presents the estimates of the latency parameters, their standard deviations (SD), and the p -values. Clearly, at 5% level of significance, only noadyn turns out to be significant for all models as far as the time to onset of grade IV VGE for uncured patients is concerned. Note that the effect of noadyn is the same for all models. Since the estimate of $\gamma_{4}$ is positive, ambulatory subjects tend to experience grade IV VGE faster. This finding is consistent with Ma.^41^

**Table 8.** Estimation results corresponding to the latency parameters for the HDSD data.

	Estimates			SD			p -value		
Parameter	SVM	Spline	Logistic	SVM	Spline	Logistic	SVM	Spline	Logistic
Age	0.0294	−0.1798	−0.1947	0.1037	0.0967	0.115	0.7767	0.0631	0.1418
TR360	0.0628	−0.2697	−0.2522	0.1744	0.1741	0.145	0.7187	0.1214	0.1632
Sex	0.3449	−0.1232	−0.2908	0.4459	0.3431	0.107	0.4392	0.7196	0.5910
Noadyn	1.3252	1.5849	1.6868	0.4081	0.3072	0.107	1.17 $\times 10^{-3}$	2.47 $\times 10^{-7}$	8.00 $\times 10^{-4}$

SVM: support vector machine; HDSD: Hypobaric decompression sickness database; SD: standard deviation.

## 5. Conclusion

The SVM has received a great amount of interest in the past two decades. It has been shown that the SVM performs well in a wide array of problems including face detection, text categorization and pedestrian detection. However, the use of the SVM in the context of cure rate models is new and not well explored. In this article, we have proposed a new cure rate model that uses the SVM to model the incidence part and a PH structure to model the latency part for survival data subject to interval censoring. The new cure rate model inherits the properties of the SVM and can capture more complex classification boundaries. For the estimation purpose, we have proposed an EM algorithm where sequential minimal optimization together with Platt scaling method are employed to estimate the uncured probabilities. In this regard, due to the unavailability of some cured statuses, we make use of a multiple imputation-based approach to generate missing cured statuses. Due to the complexity of the proposed model and the estimation method, we approximate the standard errors of the estimated parameters using non-parametric bootstrapping. Through a simulation study, we have shown that when the true classification boundary is nonlinear the proposed SVM-based mixture cure model overall performs better than the standard logistic regression-based and spline-based mixture cure models. As future research, it is of great interest to study the performance of the proposed model in the presence of high-dimensional covariates and to develop computationally efficient methods for covariate selection. It is also of great interest to extend the proposed model to accommodate a competing risks scenario.^18,60^ Furthermore, it is also possible to explore other machine learning algorithms (e.g. NN or tree-based approaches) to study more complicated cure rate models such as those that look at the elimination of risk factors.^61–66^ We are currently looking at some of these problems and we hope to report the findings in our upcoming manuscripts.

## Supplemental Material

sj-pdf-1-smm-10.1177_09622802231210917 - Supplemental material for A support vector machine-based cure rate model for interval censored data Supplemental material, sj-pdf-1-smm-10.1177_09622802231210917 for A support vector machine-based cure rate model for interval censored data by Suvra Pal, Yingwei Peng, Wisdom Aselisewine and Sandip Barui in Statistical Methods in Medical Research
