# Supplementary material for: A support vector machine-based cure rate model for interval censored data
Source: Stat Methods Med Res. 2023 Nov 8;32(12):2405–22. doi: 10.1177/09622802231210917 (PMC10710011; doi:10.1177/09622802231210917)
Supplement: sj-pdf-1-smm-10.1177_09622802231210917 - Supplemental material for A support vector machine-based cure rate model for interval censored data [file sj-pdf-1-smm-10.1177_09622802231210917.pdf]

Supplemental material for “A Support Vector Machine Based Cure Rate Model for Interval Censored Data”

A1. R code for data generation

```
# function to find the left and right end points that contain the event time

LR_int=function(y1,len1,l1){
  if(y1>0 & y1<=l1){
    a=c(.Machine$double.eps,l1)
  }else{
    k=as.integer((y1-l1)/len1)+1
    a=c(l1+((k-1)*len1),l1+(k*len1))
  }
  return(a)
}

# Data Generating Function
data.int = function(n,alpha,beta1,beta2,cens,setting){
  z1 = rnorm(n,mean=0,sd=1)
  z2 = rnorm(n,mean=0,sd=1)

  piz = rep(NA,n) # this is the uncured probability pi(z)

  if(setting==1){
    piz = (exp(0.3-(5*z1)-(3*z2))/(1+exp(0.3-(5*z1)-(3*z2))))
  }
  if(setting==2){
    piz =  (exp(0.3+(10*z1*z2)-(5*z1*z2))/(1+exp(0.3+(10*z1*z2)-(5*z1*z2))))
  }

  C = runif(n,0,cens) # censoring time
  U = runif(n,0,1)
  L = rep(NA,n) # left end point
  R = rep(NA,n) # right end point
  D = rep(NA,n) # censoring indicator
  J = rep(NA,n) # cured indicator (J=0 implies cured)
  Sp = rep(NA,n)
  S1 = rep(NA,n)

  for(i in 1:n){
    if(U[i]<= 1-piz[i]){
```

```
        L[i] = C[i]
        R[i] = 1/0
        D[i] = 0
        J[i] = 0

        Sp[i] = (1-piz[i]) + (piz[i]*exp(-((L[i]/exp(-((beta1*z1[i])+
                  (beta2*z2[i]))/alpha))^alpha)))
        S1[i] =  (exp(-((L[i]/exp(-((beta1*z1[i])+(beta2*z2[i]))/alpha))^alpha)))

      }else{
        T1 =  rweibull(1,shape=alpha,scale= exp(-((beta1*z1[i])+(beta2*z2[i]))/alpha) )
        J[i] = 1
        if(min(T1,C[i])==C[i]){
          D[i] = 0
          L[i] = C[i]
          R[i] = 1/0
          Sp[i] = (1-piz[i]) + (piz[i]*exp(-((L[i]/exp(-((beta1*z1[i])+
                    (beta2*z2[i]))/alpha))^alpha)))
          S1[i] = (exp(-((L[i]/exp(-((beta1*z1[i])+(beta2*z2[i]))/alpha))^alpha)))
        }else{
          len=runif(1,0.2,0.7)
          l=runif(1,0,1)
          ans=LR_int(T1,len,l)
          L[i]=ans[1]
          R[i]=ans[2]
          Sp[i] = (1-piz[i]) + (piz[i]*exp(-((((L[i]+L[i])/2)/exp(-((beta1*z1[i])+
                    (beta2*z2[i]))/alpha))^alpha)))
          S1[i] = (exp(-((((L[i]+L[i])/2)/exp(-((beta1*z1[i])+
                    (beta2*z2[i]))/alpha))^alpha)))
          D[i] = 1
        }
      }
    }

  return(data.frame(L,R,D,z1,z2,J,uncure=piz,Sp=Sp,S1=S1))
}
```

A2. R code for SVM-based EM algorithm

```
library(e1071)
library(caTools)
library(survival)
library(dplyr)

# EM code

em.svm.tb=function(TimeL,TimeR,Status,X,Z,offsetvar,uncureprob,uncurepred,
           beta,emmax,eps,data,testdata)
{
  dat1_0=data.frame(l=TimeL,r=TimeR)

  m <- dim(testdata[1])
  w <- Status
  n <- length(Status)
  tb1 <- Turnbull(dat1_0)
  TimeL_s=rep(NA,length(TimeL))
  TimeR_s=rep(NA,length(TimeR))

  for(j in 1:length(TimeL)){
    TimeL_s[j]=tb1$S.new[which(tb1$tau==TimeL[j])]
    if(TimeL_s[j]==0){TimeL_s[j]=.Machine$double.eps}
  }

  for(j in 1:length(TimeR)){
    if(TimeR[j]==Inf){TimeR_s[j]=0}
    else{
      TimeR_s[j] = tb1$S.new[which(tb1$tau==TimeR[j])]
      if(TimeR_s[j]==0){TimeR_s[j]=.Machine$double.eps}
    }
  }

  convergence<- 1000;i <-1

  while (convergence > eps & i < emmax){

    survival=drop((TimeL_s)^(exp((beta)%*%t(X[,-1]))))

    ## E step
    w <- Status+(1-Status)*(uncureprob*survival)/((1-uncureprob)+uncureprob*survival)
    ## M step
    multipleuncureprob=matrix(1:5*n, nrow=n,ncol=5)
    for (j in 1:n){multipleuncureprob[j,]<-rbinom(5,size = 1,prob=w[j])}
    uncureprob1<-c(1,1)
    uncureprob2<-c(1,1)
    uncureprob3<-c(1,1)
    uncureprob4<-c(1,1)
    uncureprob5<-c(1,1)
    for (j in 1:n){uncureprob1[j]=multipleuncureprob[j,1]}
```

```
for (j in 1:n){uncureprob2[j]=multipleuncureprob[j,2]}
for (j in 1:n){uncureprob3[j]=multipleuncureprob[j,3]}
for (j in 1:n){uncureprob4[j]=multipleuncureprob[j,4]}
for (j in 1:n){uncureprob5[j]=multipleuncureprob[j,5]}

for (j in 1:n){uncureprob1[j]=uncureprob1[j]*2-1}
for (j in 1:n){uncureprob2[j]=uncureprob2[j]*2-1}
for (j in 1:n){uncureprob3[j]=uncureprob3[j]*2-1}
for (j in 1:n){uncureprob4[j]=uncureprob4[j]*2-1}
for (j in 1:n){uncureprob5[j]=uncureprob5[j]*2-1}

uncureprob1<-as.factor(uncureprob1)
uncureprob2<-as.factor(uncureprob2)
uncureprob3<-as.factor(uncureprob3)
uncureprob4<-as.factor(uncureprob4)
uncureprob5<-as.factor(uncureprob5)
update_cureb<-c(1,1)
update_pred<-c(1,1)

obj<-tune(svm,uncureprob1~Z[,-1],data=data,kernel="radial",
      ranges=list(gamma=2^(-1:1),cost=2^(2:4)),
      tunecontrol=tune.control(sampling = "fix"))
bg<-obj$best.parameters[1]
bc<-obj$best.parameters[2]
mod1<-svm(Z[,-1],uncureprob1,method = "C-classification", kernel="radial",
       gamma=bg[[1]],cost=bc[[1]], probability=TRUE)
pred1<-predict(mod1,newdata=Z[,-1],probability = TRUE)
cpred1<-predict(mod1,newdata=testdata[4:5],probability = TRUE)
proba1<-attr(pred1, "probabilities")
cproba1<-attr(cpred1, "probabilities")
update_cureb1<-c(1,1)
update_pred1<-c(1,1)
for (z in 1:n){update_cureb1[z]<-proba1[z,colnames(proba1)==1]}
for (k in 1:m){update_pred1[k]<-cproba1[k,colnames(cproba1)==1]}
uncureprob1<-as.numeric(as.character(uncureprob1))

mod2<-svm(Z[,-1],uncureprob2,method = "C-classification", kernel="radial",
       gamma=bg[[1]],cost=bc[[1]],probability=TRUE)
pred2<-predict(mod2,newdata=Z[,-1],probability = TRUE)
cpred2<-predict(mod2,newdata=testdata[4:5],probability = TRUE)
proba2<-attr(pred2, "probabilities")
cproba2<-attr(cpred2, "probabilities")
update_cureb2<-c(1,1)
update_pred2<-c(1,1)
for (z in 1:n){update_cureb2[z]<-proba2[z,colnames(proba2)==1]}
for (k in 1:m){update_pred2[k]<-cproba2[k,colnames(cproba2)==1]}
uncureprob2<-as.numeric(as.character(uncureprob2))

mod3<-svm(Z[,-1],uncureprob3,method = "C-classification", kernel="radial",
       gamma=bg[[1]],cost=bc[[1]],probability=TRUE)
pred3<-predict(mod3,newdata=Z[,-1],probability = TRUE)
cpred3<-predict(mod3,newdata=testdata[4:5],probability = TRUE)
```

```
proba3<-attr(pred3, "probabilities")
cproba3<-attr(cpred3, "probabilities")
update_cureb3<-c(1,1)
update_pred3<-c(1,1)
for (z in 1:n){update_cureb3[z]<-proba3[z,colnames(proba3)==1]}
for (k in 1:m){update_pred3[k]<-cproba3[k,colnames(cproba3)==1]}
uncureprob3<-as.numeric(as.character(uncureprob3))

mod4<-svm(Z[,-1],uncureprob4,method = "C-classification", kernel="radial",
      gamma=bg[[1]],cost=bc[[1]],probability=TRUE)
pred4<-predict(mod4,newdata=Z[,-1],probability = TRUE)
cpred4<-predict(mod4,newdata=testdata[4:5],probability = TRUE)
proba4<-attr(pred4, "probabilities")
cproba4<-attr(cpred4, "probabilities")
update_cureb4<-c(1,1)
update_pred4<-c(1,1)
for (z in 1:n){update_cureb4[z]<-proba4[z,colnames(proba4)==1]}
for (k in 1:m){update_pred4[k]<-cproba4[k,colnames(cproba4)==1]}
uncureprob4<-as.numeric(as.character(uncureprob4))

mod5<-svm(Z[,-1],uncureprob5,method = "C-classification", kernel="radial",
      gamma=bg[[1]],cost=bc[[1]],probability=TRUE)
pred5<-predict(mod5,newdata=Z[,-1],probability = TRUE)
cpred5<-predict(mod5,newdata=testdata[4:5],probability = TRUE)
proba5<-attr(pred5, "probabilities")
cproba5<-attr(cpred5, "probabilities")
update_cureb5<-c(1,1)
update_pred5<-c(1,1)
for (z in 1:n){update_cureb5[z]<-proba5[z,colnames(proba5)==1]}
for (k in 1:m){update_pred5[k]<-cproba5[k,colnames(cproba5)==1]}
uncureprob5<-as.numeric(as.character(uncureprob5))

for (z in 1:n){update_cureb[z]<-(update_cureb1[z]+update_cureb2[z]+update_cureb3[z]+
                              update_cureb4[z]+update_cureb5[z])/5}
for (k in 1:m){update_pred[k]<-(update_pred1[k]+update_pred2[k]+update_pred3[k]+
                              update_pred4[k]+update_pred5[k])/5}

# latency part
Q1 = function(par=c(b1,b2)){
  bb = c(par[1],par[2])
  S.L = drop((TimeL_s)^(exp((bb)%*%t(X[,-1]))))
  S.R = drop((TimeR_s)^(exp((bb)%*%t(X[,-1]))))
  out = sum((1-Status)*w*log(S.L)) + sum(Status*log(S.L-S.R))
  return(-out)
}

update_latency = optim(par=c(beta[1],beta[2]),fn=Q1,method="Nelder-Mead")$par

convergence<-sum(c(mean(update_cureb)-mean(uncureprob),update_latency-beta)^2)

uncureprob <- update_cureb
uncurepred <- update_pred
```

```
    beta <-  update_latency

    i <- i+1
  }

  S1 = drop((TimeL_s)^(exp((beta)%*%t(X[,-1]))))
  Sp = (1-uncureprob)+(uncureprob*S1)
  em.svm <- list(latencyfit= beta,Uncureprob=uncureprob,Uncurepred=uncurepred,
                Sp=Sp,S1=S1,tau=convergence, Mod1=mod1, Mod2=mod2,
                Mod3=mod3, Mod4=mod4, Mod5=mod5)
}
```
